# Next-generation sequencing analysis of receptor-type tyrosine kinase genes in surgically resected colon cancer: identification of gain-of-function mutations in the RET proto-oncogene

**DOI:** 10.1186/s13046-018-0746-y

**Published:** 2018-04-17

**Authors:** Duarte Mendes Oliveira, Katia Grillone, Chiara Mignogna, Valentina De Falco, Carmelo Laudanna, Flavia Biamonte, Rosa Locane, Francesco Corcione, Massimiliano Fabozzi, Rosario Sacco, Giuseppe Viglietto, Donatella Malanga, Antonia Rizzuto

**Affiliations:** 1 Department of Experimental and Clinical Medicine, University Magna Graecia of Catanzaro, Campus Salvatore Venuta -Viale Europa, Catanzaro, 88100 Italy; 2 Department of Health Sciences, University Magna Graecia of Catanzaro, Campus Salvatore Venuta - Viale Europa, Catanzaro, 88100 Italy; 3 Department of Medical and Surgical Sciences, University Magna Graecia of Catanzaro, Campus Salvatore Venuta - Viale Europa, Catanzaro, 88100 Italy; 4 Department of Molecular Medicine and Medical Biotechnologies, University Federico II, Naples, Italy; 5 UOC Chirurgia Generale, Azienda Ospedaliera dei Colli, Naples, Italy

**Keywords:** Next-generation sequencing, Receptor-type tyrosine kinases, Colon cancer, RET proto-oncogene

## Abstract

**Background:**

Improvement in genetic characterization of Colon Cancer (CC) patients is required to propose new potential targets, since surgical resection coupled to chemotherapy, presents several limits such as cancer recurrence and drug resistance. Targeted therapies have more efficacy and less toxicity than standard treatments. One of the most relevant cancer-specific actionable targets are receptor tyrosine kinases (RTKs) whose role in CC need to be better investigated.

**Methods:**

We have analysed 37 CC patients using the Ion AmpliSeq™ Comprehensive Cancer Panel (CCP). We have confirmed the somatic nature of RET variants through Sanger sequencing and assessed RET activation status and protein expression by immunofluorescence and western-blot analyses. We have used RET mutant expression vectors to evaluate the effect of selected mutations in HEK293 cells by performing proliferation, migration and clonogenic assays.

**Results:**

Among the 409 cancer-related genes included in the CCP we have focused on the RTKs. Overall, we have observed 101 different potentially damaging variants distributed across 31 RTK genes in 28 patients. The most frequently mutated RTKs were FLT4, ROS1, EPH7, ERBB2, EGFR, RET, FGFR3 and FGFR4. In particular, we have identified 4 different somatic variants in 10% of CC patients in RET proto-oncogene. Among them, we have demonstrated that the G533C variant was able to activate RET by promoting dimer formation and enhancing Y1062 phosphorylation. Moreover, we have demonstrated that RET G533C variant was able to stimulate anchorage-dependent proliferation, migration and clonogenic cell survival. Notably, the effects induced by the RET G533C variant were abolished by vandetanib.

**Conclusions:**

The discovery of pathogenic variants across RTK genes in 75% of the CC patients under analysis, suggests a previously underestimated role for RTKs in CC development. The identification of a gain-of-function RET mutation in CC highlights the potential use of RET in targeted therapy.

**Electronic supplementary material:**

The online version of this article (10.1186/s13046-018-0746-y) contains supplementary material, which is available to authorized users.

## Background

Targeted therapy of cancer has more efficacy and less toxicity than conventional chemotherapy because it is directed against cancer-specific molecules and/or signalling pathways. So far the most relevant cancer-specific actionable targets are protein tyrosine kinases (PTKs) [1]. PTKs regulate several cellular processes that include cell proliferation, cell death, motility and invasion [2]. When subverted, these processes may give origin to cancer [2].

The catalogue of genes encoding PTKs within the human genome has been updated to 90 genes, of which 58 are receptor type PTKs and 32 are non-receptor type PTKs [3].Genes encoding PTKs - both receptor and non-receptor - can be oncogenically activated through a variety of mechanisms that include gain-of-function mutations as in the case of EGFR in lung cancer and KIT in GIST [4–7], translocations as in the case of BCR-ABL in CML, ALK in lung cancer and RET in thyroid, lung and colorectal cancer [8–10] and amplifications as in the case of ERBB2 in breast cancer and EGFR in lung, HSNCC and colon cancer [11–15].

In any case, the constitutive signalling from PTKs leads to aberrant activation of downstream pathways that promote continued cell growth and survival. Cancer cells are particularly sensitive to intracellular pathways that are improperly hyperactive, a phenomenon known as oncogene/pathway addiction. This phenomenon provides a unique therapeutic opportunity to block cancer by inhibiting PTK activity [16].

Colorectal cancer (CRC) is one of the most commonly diagnosed and lethal cancers worldwide, with 1.4 million new cases and 690,000 deaths in 2012 [17]. Prognosis of CRC patients depends on cancer stage at diagnosis. Detection of CRC at early stage may confer a 90% 5-year survival rate, compared to 12% if distant metastasis has occurred.

Current therapy for colon cancer patients include surgical resection coupled to chemotherapy [18]. In advanced disease, either FOLFOX (oxaliplatin/5-fluorouracil [5-FU]/leucovorin [LV]) or FOLFIRI (irinotecan/LV/5-FU) are typical regimens for first-line chemotherapy, but cancer recurs in many patients [19]. Targeted therapy is suggested for patients with metastatic disease [18]. In these patients the use of monoclonal antibodies to epidermal growth factor receptor (EGFR) (cetuximab, panitumumab) and/or to vascular endothelial growth factor (VEGF) (bevacizumab) has increased the median survival beyond 20 months [20]. However, the responses to EGFR-targeted antibodies are relatively low, with improvement in survival rates lasting only few months [21].

In addition, the efficacy of anti-EGFR antibodies is limited to patients with tumors lacking mutations in effectors downstream EGFR such as KRAS, BRAF, PIK3CA and PTEN [22]. Also the patients that initially respond to the anti-VEGF therapy [23, 24] will eventually show evidence of resistance to anti-angiogenic therapy [25].

Clearly, we need to improve the molecular characterization of tumors from CRC patients in order to identify the molecular alterations inside cancer cell genomes that drive cancer growth and determine the inefficacy of anti-EGFR/VEGF targeted therapies. In a previous work we have reported the analysis of 37 colon cancer patients using the Ion AmpliSeq™ Comprehensive Cancer Panel on the Ion Proton platform (Oliveira et al., under review). In this manuscript, we have focused the analysis on the role of genes encoding RTKs as potentially targeting genes in colon cancer. The discovery of pathogenic variants in this gene family in the majority of colon cancer patients suggests a previously underestimated role for RTKs in the development of colon cancer and thus the possibility to treat those patients with specific RTK inhibitors. In particular, the identification of gain-of-function RET variants in a subset of colon cancer, as described here, has led to the identification of a small subset of patients potentially treatable with RET inhibitors.

## Methods

### Tumor samples

Patient accrual was conducted according to Institutional Review Board of the Mater Domini/University Magna Graecia (Catanzaro, Italy). The study was approved by the Institutional Review Board of the Mater Domini/University Magna Graecia in the meeting of May 21st 2014. Tumor, normal mucosa and peripheral blood samples were obtained from patients referring to General Surgey Unit of University Hospital Magna Graecia of Catanzaro (Catanzaro, Italy), who underwent surgical resection for colon cancer since January 2013. Biopsies were immediately snap frozen and stored at − 80 °C. In all cases diagnosis was confirmed by reviewing hematoxylin/eosin-stained slides.

### Patients’ demographics

General demographic information, clinical findings, surgical treatment, histo-patological examination and follow-up data were collected for each patient. Detailed documentation of the histo-pathological findings allowed classification according to the current edition of UICC [26]. None of the patients received chemotherapy or radiation therapy prior to surgery. See Additional file 1 for complete characteristics of patients. Briefly, among the 37 patients, 13 were women and 24 were males. The mean age was 68 years old (range 47–84). Stage was known for 36 of the 37 patients: 7 patients had stage I disease, 13 patients had stage II disease, 12 patients had stage III disease and 4 patients had stage IV disease. Grade was known for 35 out of 37 patients: 26 patients had tumors that were graded G2 and 9 patients had tumors that were graded G3. Four patients presented distant metastasis.

### Next generation sequencing

Sequencing was performed with the Ion AmpliSeq™ Comprehensive Cancer Panel on the Ion Proton system (ThermoFisher Scientific, MA, USA) starting from 40 ng of DNA and following standard protocols. Primary bioinformatic analysis of data is described in a previous manuscript (Oliveira et al., under review). We set an average mean coverage of > 300, a coverage of > 40 for each variant and variant frequency > 5%. To further filter the number of non-pathogenic variants identified in the study the following criteria were used: i) variants with quality score ≤ 30 were excluded, ii) variants that were not annotated as pathogenic or likely pathogenic by SIFT and Polyphen2 algorithms were excluded, iii) variants detected in both normal and tumor samples were excluded. Finally, manual observation of the variants using the Integrated Genomic Viewer (IGV – Broad Institute) was performed to exclude inconclusive variants.

### Sanger sequencing

DNA obtained from matched normal and tumor samples was amplified using specific primers. The purified products were sequenced using BigDye terminator v3.1 (Applied Biosystems, Foster City, CA) with ABI 3100 Genetic Analyser (Applied Biosystems, Foster City, CA).

### RNA extraction and reverse-transcription

Tumors obtained at surgery were snap frozen in liquid nitrogen and conserved at − 80 °C. RNA was prepared using Trizol (ThermoFisher Scientific, MA, USA) and purified using RNeasy mini-eluate clean-up kit (QIAGEN Inc. CA, USA). cDNA was retro-transcribed with random hexamers using SuperScript®III First-Strand Synthesis System (ThermoFisher Scientific, MA, USA).

### Immunostaining

Deparaffinized 4-μm sections were hydrated in decreasing ethanol gradient solutions and rinsed in wash solution (TBST, 0.05 mol/L Tris Buffered Saline with Tween20). Antigen retrieval was performed with citrate buffer pH 6 for antibodies to RET and phospho-RET Y905, and pH 9 for antibody to phospho-RET Y1062, respectively, for 20 min at 98 °C followed by washing in TBST. Sections were incubated with antibodies against RET (rabbit monoclonal antibody anti-RET, 1:250 dilution, #14556 Cell Signaling Technology, Danver, MA), phospho-RET Y905 (rabbit polyclonal antibody anti-pRET, 1:100 diluition, #3221 Cell Signaling Technology) or phospho-RET Y1062 (rabbit polyclonal antibody anti p-RET, 1:100 dilution) [27] for 60 min, followed by incubation with secondary Alexa Fluor 488F fragment of goat anti-rabbit IgG (H + L) antibody; 1:500 dilution (ThermoFisher Scientific, MA, USA #A11070) for 45 min. Cells were counterstained with DAPI,1:500 dilution, (ThermoFisher Scientific, MA, USA), mounted using anti-fade mounting medium #53023 (DAKO, Glostrup, Denmark) and observed at Fluorescence Microscopy (Leica Microsystems, Wetzlar Germany).

### Protein extraction and immunoblot

Protein extracts were prepared with lysis buffer containing 50 mM HEPES pH 7.5, 5 mM EDTA, 250 mM NaCl, 1 mM dithiothreitol, 0.5% Nonidet P40, 1 mM Na_3_VO_4_, 1 mM NaF supplemented with 10 μg of aprotinin/ml, 10 μg of leupeptin/ml, 1 mM PMSF and a mix of protease inhibitors (SIGMA*FAST* protease inhibitor Tablets for general Use; Sigma-Aldrich St. Louis, MO, USA). Lysates were centrifuged at 13,000 rpm for 30 min at 4 °C and the supernatants were collected. Protein concentration was estimated with a modified Bradford assay (Bio-Rad Laboratories, Berkeley, CA, USA). Western blot analysis was carried out by standard methods.

To detect RET dimers, electrophoresis was carried out on a 6% SDS–PAGE in non-reducing conditions. In this case proteins extracts were prepared in the same lysis buffer as above devoid of dithiothreitol, and with sample loading buffer devoid of 2-mercaptoethanol. Samples were not heated upon loading.

Proteins were revealed by enhanced chemiluminescence detection using Clarity™ Western ECL Substrate (Bio-Rad Laboratories, Berkeley, CA, USA). The antibodies used in this study were: anti-RET (E1N8X, #14556), anti-phospho-RET, (#3221), anti-phospho-ERK1/2 (#9101), anti-ERK1/2 (#9107) purchased from Cell Signaling Technology, Danver, MA, USA. The anti-phospho-RET Y1062 used in this study has been previously described [27].

### Construction of RET mutants expression vectors

RET mutants were obtained by site-specific mutagenesis of RET51-WT construct (pBabe vector encoding for the proto-RET gene long isoform). The RET51-C634R plasmid, used as positive control, is described elsewhere [28]. RET51-G533C and RET51-P1047S were obtained by site-directed mutagenesis of RET51-WT using an in vitro oligonucleotide mutagenesis system (Quik-Change XL site-directed mutagenesis; Agilent Technologies, CA, USA). Plasmid DNA was extracted using the QIAGEN Plasmid Maxi Kit (QIAGEN CA, USA) as suggested by the supplier. The presence of the specific mutations was verified by DNA direct sequencing. Mutant plasmids were sequenced entirely to exclude the presence of additional mutations.

### Transfections and colony formation assay

Human HEK293T cells were maintained in Gibco™ DMEM with 10% FBS and 1% penicillin/streptomycin (ThermoFisher Scientific, MA, USA). Empty pBABE vector and/or plasmids encoding RET wild type or mutant alleles were transiently transfected using Lipofectamine 3000 (ThermoFisher Scientific, MA, USA) according to the manufacturer’s instructions. Forty-eight hours after transfection, cells were trypsinized and plated into 100-mm plates. Transfected cells were selected with 0.75 μg/ml puromycin for 1 week until all cells in the control plates were dead. Then cells were trypsinized, counted and 5000 cells were plated into 60-mm plates. Cells were kept in DMEM with 10% bovine serum. The culture medium was changed every 3–4 days. Approximately 10 days after transfection, cells were stained by crystal violet. The colony formation experiments were repeated three times.

### MTT assay

Stably transfected cells expressing RET wild type and mutant alleles were seeded in 96 wells round bottom plates (1000 cells/well). After 24 h, 10 μl of MTT [3-(4,5-dimethylthiazol-2-yl)-2,5-diphenyltetrazolium bromide]; (Sigma-Aldrich St. Louis, MO, USA) was dissolved at the concentration of 5 mg/ml in warm assay medium was added to cells at the indicated time points (24 h, 48 h, 72 h and 96 h) to yield a final assay volume of 110 μl/well. Plates were incubated for 4 h at 37 °C and 5% CO_2_. After incubation, supernatants were removed, and 100 μl of isopropanol was added. Plates were placed on an orbital shaker for 5 min, and the absorbance was recorded at 570 nm. Triplicates were made for each time point and two independent experiments were conducted. Statistical analysis was performed by two-way ANOVA and Dunnett’s multiple comparison test with GraphPad Software (La Jolla, CA, USA).

### Wound healing assay

Stably HEK293 cells expressing RET wild type or mutant alleles were seeded in a 60 mm plates and cultured until reaching 90% confluence*.* A micropipette tip (200 μl) was used to make a scratch and simulate a wound. Cells were monitored after 0 h, 24 h and 48 h from the scratch and images of wound healing were captured (magnification of 10X) using the Leica DFC420 C and Leica Application Suite Software (Leica Microsystems, Wetzlar Germany). Subsequently, cell migration was quantified by measuring the wound opening area with ImageJ64 software.

## Results

### Mutations in genes encoding RTKs in colon cancer

In a previous work, we have reported the analysis of 37 colon cancer patients using the Ion AmpliSeq™ Comprehensive Cancer Panel (CCP) (Oliveira et al., under review). In that work, patients who had undergone surgery for colon cancer at the General Surgery Unit of University Magna Graecia of Catanzaro in the years 2013–2015 were studied. Complete demographic and clinical information of patients are reported in Additional file 1. DNA was extracted from matched normal and pathological tissues and subjected to NGS analysis on the Ion Torrent platform using the CCP (ThermoFisher Scientific, MA, USA), that provides complete exon coverage of the 409 most important cancer-associated genes.

As indicated in Oliveira et al., (under review), the sequencing performance achieved was: 13 × 10^6^ mean number reads/sample, 791 (range 102.5–2656) mean sequence coverage depth of targeted exonic regions of the 409 genes analysed, with median uniformity of sequenced genes 97% (range 0.7–0.99). Common germ-line variants were removed by filtering sequentially through a pool of peripheral blood samples available from some patients (*n* = 12), the dbSNP141 and the 1000 Genomes Project data sets. Variants were functionally annotated using the algorithms Sift and Polyphen2 to predict their pathogenicity.

In this manuscript, we have re-analysed the data generated in the manuscript from Oliveira et al., focusing on genes encoding receptor-type tyrosine kinase (RTK). The number of RTK genes contained in the Comprehensive Cancer Panel that were object of the present study is 31. Overall, upon analysis of the data reported in Oliveira et al. we have observed 101 different potentially damaging variants distributed across 31 RTK genes. See Additional file 2 for a general summary of the analysis. We have found that 28 patients out of 37 presented variants in at least one gene encoding RTK, with the remaining 9 patients showing no variant in any of the 31 RTK genes. The mean number of mutated RTK genes/patient was 3.4 (range 1–13). Of the 28 samples that presented mutations in RTK genes, 8 patients had only one mutated gene, 6 had two different mutated genes and 14 presented 3 or more mutated genes. See Additional file 3 for patient-by-patient analysis of the results. Among RTK genes that presented variants, we observed variants in FLT4 (G1131S, R104Q, E1052K, D1003N, D728N, P707L, R362L, R282*, Q213*, G180R, P138S, E36* and 3 frameshifts) in 10 patients, ROS1 (S277Y, P437L, S653F, T804 N, Q1127*, G1709C and QG1708HC) in 7 patients, EPHA7 (D839G, P805L and P278S) in 6 patients, RET (R77C, P270L, G533C, P1047S) in 4 patients and MET (A320V, R988C) in 2 patients. In addition, ERBB2 variants (S310F, W482C, Q533*, T631A, S819F, A1216D) were detected in 6 patients, EGFR (R669*, W731*, D807E, L933P) and ERBB4 (R983K, M977 V, R847H, T244I) variants were detected in 4 patients, and ERBB3 variants (T389I, V850 M) were detected in 2 patients. FGFR1 (D784E, A131V) and FGFR2 (G302 K, L104P) variants were detected in 2 patients, whereas FGFR3 (D320N, A352E, A571V, T653I) and FGFR4 variants (R130C, KE144RG, F859 L, T912I) were detected in 4 patients. Eight tumors presented variants in only one RTK gene (FLT4, EGFR, ERBB4, ROS1, and EPHA7), which suggests that these variants can represent driving mutations. Accordingly, the variants detected in the EGFR, ERBB4 and FLT4 were present in the COSMIC database. Conversely, co-occurrence of variants in different genes encoding RTKs was frequent, occurring in 20 tumors out of 37 analysed (54%). Co-occurrence of variants included 2 different genes (FGFR4/NTRK1, ERRB3/FLT3, ALK/ERBB4, EPHA3/EPHA7, EPHB4/ROS1, and FLT4/LTK) in 6 cases and three or more genes in the remaining 14 cases. Notably, some samples showed variants in numerous genes encoding RTK (CC27, 11 genes; CC34, 13 genes; CC12, 8 genes).

### Identification of RET mutations in colon cancer by NGS and validation of NGS results

Among the RTKs showing variants identified in this study, we decided to biologically validate the variants identified in the gene encoding the RET receptor. The reasoning for this choice was that, at difference with what happens in thyroid and lung cancer, the involvement of RET proto-oncogene to the development of colon cancer is unclear. Variants in the RET gene were identified in four colon cancer patients (patients CC12, CC20, CC25 and CC33), as indicated in Additional file 2.

In the present study, we have identified a C/T transition that led to the replacement of P1047 with S (P1047S) in the cytoplasmic tail of RET in patient CC12, a C/T transition that led to the replacement of P270 with L (P270L) in the RET extracellular domain in patient CC33, an A/T transversion that led to the replacement of G533 with C (G533C) in the cysteine-rich domain of RET protein in patient CC20 and a G/A transition that led to the replacement of R77 with C (R77C) in the RET extracellular domain in patient CC25.

As to the technical aspects of NGS, patient CC12 showed total mean coverage of 1990, coverage of the variant of 45 and frequency of the variant 49%, patient CC33 showed total mean coverage of 789, coverage of the variant of 349 and frequency of the variant 5.9%, patient CC20 showed total coverage of 728, coverage of the variant of 442 and frequency of the variant 20.4% and patient CC25 showed total mean coverage of 761, coverage of the variant of 89 and frequency of the variant 30.5%. Raw data showing the detection of the different RET mutations by NGS are contained in Additional file 4.

**Fig. 1:**
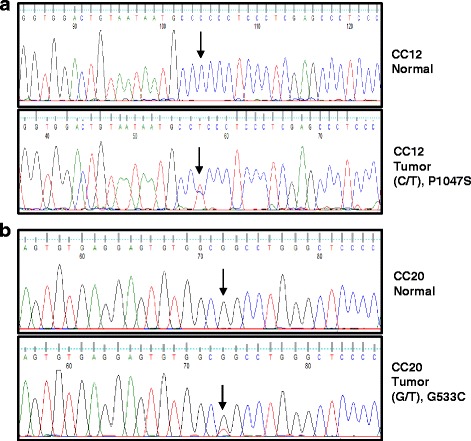
RET is somatically mutated in colon cancer samples. **a**. DNA Sanger sequencing of normal mucosa and the corresponding tumor tissue of patient CC12 [exon 12 (C/T)] (**b**). DNA Sanger sequencing of normal mucosa and the corresponding tumor tissue of patient CC20 [exon 7 (G/T)]

Interestingly, mutation G533C is located in the RET extracellular domain and consists in the replacement of a glycine amino acid with a cysteine as often observed in families with familial medullary thyroid carcinoma (FMTC) or Multiple Endocrine Neoplasia (MEN) 2A. Notably, the G533C substitution was identified in patients with FMTC [29], suggesting that it represents a gain-of-function mutation. For the complete list of mutated genes in the patient harboring the G533C substitution see Additional file 5. First we confirmed the results obtained through NGS by traditional Sanger sequencing. We performed DNA sequencing of RET exons on matched normal and cancer samples to confirm the presence of the specific mutations identified by NGS (See Fig. 1). Among the 4 different RET mutants identified in colon cancer patients we excluded the P270L variant because it represented less than 10% of the cell population and the R77C since it has been observed in a case of Hirschsprung’s disease [30]. Conversely, variants G533C and P1047S were included in the subsequent analysis. We found that in the two patients, mutations were cancer-specific and were not found in the corresponding normal tissue. These findings led us to exclude the presence of germ-line mutations associated with inherited diseases such as FMTC, MEN2 type 2A, MEN2 type 2B and/or Hirschsprung’s disease. Conversely mutations were somatically acquired and were apparently selected for within the bulk of tumor cells since they represented 20–49% of RET alleles, respectively. We analysed RET expression in the tumors and in the corresponding normal mucosa from patients CC12 and CC20 where RET mutations were identified.

**Fig. 2:**
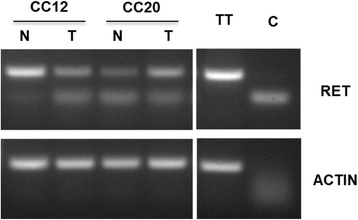
Semi-quantitative RT-PCR analysis of RET expression in colon cancer samples. Semi-quantitative RT-PCR was performed to investigate RET expression in matched normal (N) and tumor (T) samples in patients CC12 and CC20. Actin mRNA was used as control for RNA integrity and quantity. TT cells were used as positive control. Sample without input RNA was used as negative control (C)

**Fig. 3:**
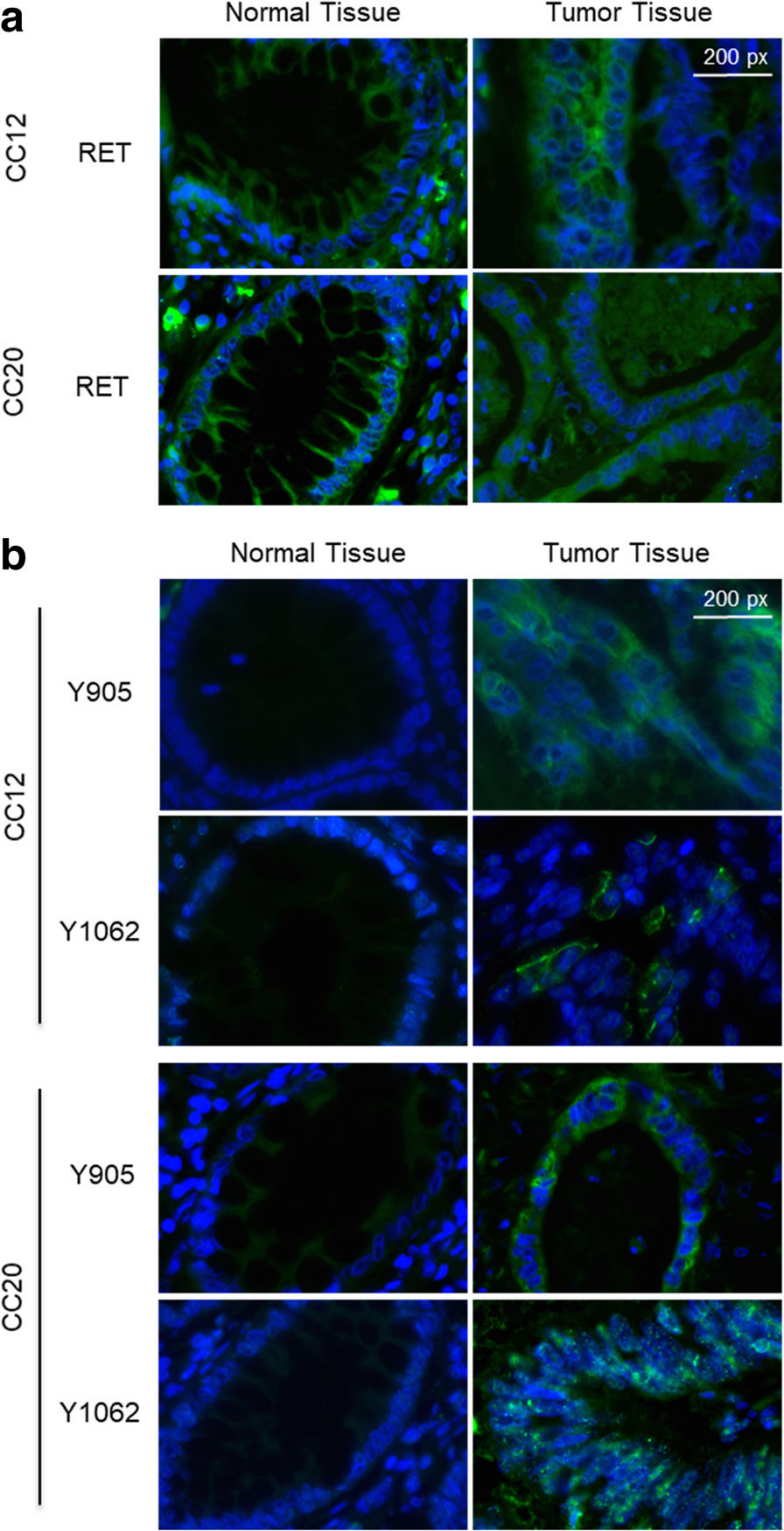
Immunofluorescence analysis of RET expression and phosphorylation in colon cancer samples. **a**. Immunofluorescence analysis of RET expression in normal and tumor tissue samples from patients CC12 and CC20 shown as merged images of green (RET staining) and blue (DAPI staining). Magnification 63X. Scale bar, 200px. **b**. Immunofluorescence analysis of RET phosphorylation at Y905 and Y1062 in normal and tumor tissue samples from patients CC12 and CC20 shown as merged images of green (phospho-RET staining) and blue (DAPI staining). Magnification 63X. Scale bar 200px

The expression of RET in the samples harbouring RET mutations was evaluated by semi-quantitative reverse transcriptase-polymerase chain reaction (RT-PCR) (Fig. 2) and by in situ immunofluorescence analysis (Fig. 3a). RT-PCR analysis revealed the presence of a RET transcript in normal mucosa and the corresponding cancer in all patients analysed. The detection of RET transcript by RT-PCR mRNA was corroborated by analysis of the RET protein in normal and cancerous tissues by immunofluorescence. Expression of RET protein and the levels of its phosphorylation at residues Y905 and Y1062 were assessed in colon cancer and the corresponding normal mucosa of patients CC12 and CC20 using immunofluorescence.

Tissues were scored as positive when showing uniform, intense cytoplasmic labelling with marked accentuation of membranes, and as negative or weakly positive when labelling was a faint cytoplasmic staining with few positive cells interspersed among the majority of negative cells. We found that normal colon mucosa of the two samples analysed were moderately positive for RET expression (see Fig. 3a).

Similarly, we found that the corresponding cancerous tissues of patients CC12 and CC20 retained expression of RET at levels that were comparable with normal mucosa. Moreover, tumor samples harbouring RET mutations showed positivity for phospho-Y905 and phospho-Y1062 residues while the normal mucosa samples were either negative or presented weak positivity (see Fig. 3b). These results suggest that, at least in the case of some RET mutants, the expression of the receptor is not impaired by selective pressure for loss of RET expression.

### RET activating mutations identified in colon cancer patients increase anchorage-dependent cell proliferation and clonogenic cell survival

We investigated whether the RET variants identified in colon cancer patients are able to stimulate anchorage-dependent proliferation in HEK293 cells. This cellular system has already been successfully used to investigate the oncogenic characteristics of multiple oncoproteins [31]. To this aim, the G533C and P1047S RET variants were generated by site-specific mutagenesis of pBABE-puro plasmid carrying the cDNA encoding human wild type RET (RET-G533C, RET-P1047S and RET-WT, respectively).

HEK293 cells were transfected with empty vector (HEK293-C) or transfected with plasmids carrying wild type or mutant RET (HEK293-RET-WT, HEK293-RET-G533C and HEK293-RET-P1047S, respectively). Multiple clones of HEK293-C, HEK293-RET-WT, HEK293-RET-G533C and HEK293-RET-P1047S cells from transfection experiments were expanded and/or frozen for further studies. HEK293-RET-C634R cells were used as positive control.

**Fig. 4:**
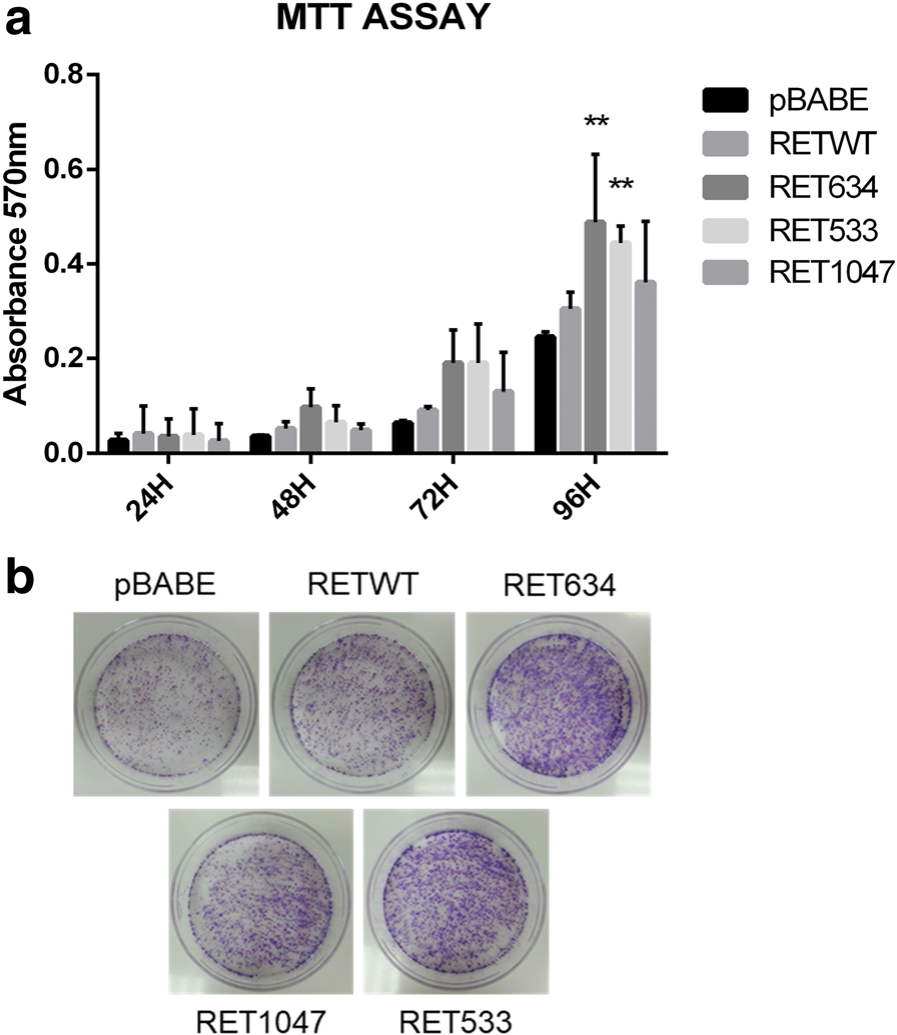
Analysis of the biological effects exerted by RET-G533C and RET-P1047S mutants. Plasmids encoding wild type or mutant RET variants (G533C and P1047S) were transfected into HEK293 cells and selected in puromycin. **a**. MTT assay was performed at different time points (24 h, 48 h, 72 h and 96 h) using empty pBabe plasmid as negative control and plasmid encoding RET C634R mutant as positive control. Values are shown as bar graphs and all results are the average of two independent experiments performed in triplicate, Error bars s.d.; *n* = 6; **p < 0.01; ***p < 0.001 compared with control. **b**. Colony formation assay was performed with HEK293 cells transfected with plasmids encoding wild type or mutant RET variants (G533C and P1047S). We used empty pBabe plasmid as negative control and plasmid encoding RET C634R mutant as positive control. Similar results were observed in three independent experiments

Analysis of cell proliferation by MTT demonstrated that, similar to HEK293-RET-C634R cells, HEK293-RET-G533C duplicated with an increased rate in monolayer compared with HEK293-C (*p* < 0.01). Conversely, HEK293-RET-WT and HEK293-RET-P1047S cells showed a more limited increase, being intermediate between negative (HEK293-C) and positive controls (HEK293-RET-C634R), see Fig. 4a.

Similar results were obtained with colony assay experiments. Also in this type of assay, HEK293-RET-G533C cells showed greater proliferative potential in clonogenic assays than HEK293-C cells, generating more colonies than control cells. Similarly, HEK293-RET-WT and HEK293-RET-P1047S cells showed an intermediate behaviour (Fig. 4b). These results indicate that RET mutant G533C, but not wild type or P1047S RET mutants expressed at similar level, is able to stimulate anchorage-dependent proliferation of human epithelial cells in culture.

**Fig. 5:**
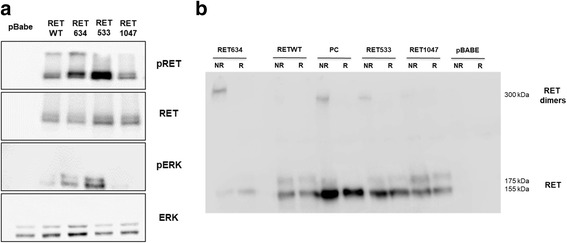
Analysis of the biochemical effects exerted by RET-G533C and RET-P1047S mutants. Immunoblot analysis of RET expression and activity in transfected HEK293 cells (**a**). Lysates from transfected HEK293 cells were analysed by immunoblot with anti-phosphoY1062 or anti-RET antibody. The activation status of the MAPK pathway was assessed with anti phospho-Y202T204 ERK1/2 antibody. Anti-ERK1/2 antibody was used for normalization. **b**. Immunoblot analysis of RET dimers in transfected HEK293 cells. Samples were prepared under non-reducing (NR) or reducing (R) conditions to detect RET dimer formation and loaded onto SDS-PAGE in non-reducing conditions. Half the total amount of protein lysate was loaded in the case of RET634

Subsequently, immunoblot analysis was performed to check for RET expression and for the activation status of MAPK pathway in transfected HEK293 cells (Fig. 5). Lysates from transfected HEK293 cells and derivatives were analysed by immunoblot with anti-phosphoY1062 or anti-RET antibody. In agreement with the data from MTT and colony assay experiments, the G533C RET mutant showed increased RET phosphorylation than wild type RET or P1047S mutant (Fig. 5a). Subsequently, we assessed the activation status of critical signalling pathways that function downstream RET [32], the MAPK pathway. The activity of the MAPK pathway was assessed with anti phospho-Y202/T204 ERK1/2 antibody (Fig. 5a). Anti-total ERK1/2 antibody was used for normalization. We found that the adoptive expression of RET mutant G533C, at difference with wild type or P1047S RET, induced a marked increase in the activation of MAPK pathway. Finally, since the mutation carried by the active mutant involved a cysteine residue in the extracellular domain, we investigated whether this amino acidic change facilitated the formation of RET dimers. To detect RET dimer formation we separated cell lysates from transfected HEK293 cells by SDS-PAGE under either non-reducing or reducing condition. As shown in Fig. 5b, we found that RET mutants G533C was particularly efficient in generating RET dimers in transfected HEK293 cells comparable to the positive control (PC), suggesting that the oncogenic mechanism whereby RET mutant G533C contribute to colon cancer development is through an increase in the amount of covalently linked dimers induced by the presence of the unpaired cysteine induced by the mutation.

**Fig. 6:**
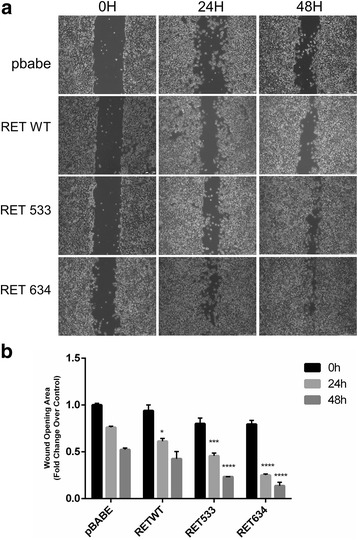
Effect of RET-G533C on cell migration. **a**. Wound healing assay of HEK293 cells expressing different RET mutants or empty pBABE vector. Cells were scratch-wounded with a micropipette tip (200 μl). Images of cellular migration were taken at times 0 h, 24 h and 48 h using the Leica DFC420 C and Leica Application Suite Software. Magnification, 10X. **b**. Wound healing was quantified by the ImageJ 64 software using the area of the wound of cells expressing empty pBabe vector at T0 as reference value. Final results represent mean ± SD of three independent experiments and are indicated as fold change over the control in terms of reduction of the wound. Statistical significance was evaluated by Two-Way ANOVA (with multiple comparison Tukey’s test) confronting at each time point the different conditions and indicating the difference over the control (***p < 0.001; ****p < 0.0001)

We also found that RET-G533C mutant stimulated cell migration in vitro (Fig. 6). We investigated the effects of the RET-G533C mutant on the migration capability of HEK293 cells by using wound healing assay (Fig. 6a). RET-WT and RET-C634R were used as controls. The results of a triplicate set of independent experiments demonstrated that RET-G533C mutant enhanced migration of HEK-293 cells of about 70% at 48 h at difference with RET-WT, which was much less efficient in promoting migration (Fig. 6b).

### Cells expressing the RET G533C mutant are sensitive to treatment with the RET specific inhibitor vandetanib.

**Fig. 7:**
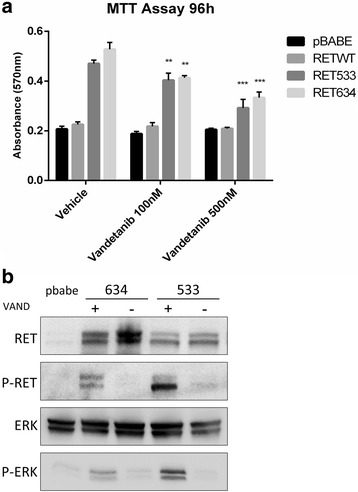
The RET-G533C mutant is inhibited by vandetanib. **a**. MTT assay was performed in the presence of two different concentrations of vandetanib. HEK293 cells expressing pBabe were used as negative control and HEK293 cells expressing RET-C634R mutant were used as positive control. Values are shown as bar graphs and all results are the average of experiments performed in triplicate. Statistical significance compared with vehicle was evaluated by Two-Way ANOVA (with multiple comparison Dunnet’s test) (*n* = 3; **p < 0.01; ***p < 0.001). **b**. Immunoblot analysis of RET/MAPK pathway (with anti-phosphoY1062 RET, anti-RET, anti phospho-Y202T204 ERK1/2, anti-ERK1/2 antibodies) of HEK293 cells transfected with RET-G533C mutant treated with vehicle or vandetanib (500 nM) for 2 h. HEK293 cells transfected with RET-634 mutant were used as control

Finally, we determined whether the RET inhibitor vandetanib was able to abolish the effects exerted by RET-533C mutant in transfected HEK293 cells. Treatment of HEK393 cells expressing RET mutants with vandetanib induced a dose-dependent reduction (almost 50% using 500 nM Vandetanib) in viable cell number of HEK293-RET-G533C (and HEK293-RET-C634R) cells, demonstrating growth inhibitory activity in vitro (Fig. 7a). Similarly, subsequent immunoblot analysis demonstrated that vandetanib markedly reduced phosphorylation of RET Y1062 and of ERK1/2 Y202/T204 in HEK293-RET-G533C and HEK293-RET-C634R cells (Fig. 7b).

## Discussion

In this manuscript, we have re-analysed NGS data generated on a cohort of Italian patients affected by colon cancer to identify variants in genes encoding RTKs. The main findings of this analysis were: i) somatic variants were detected in 31 RTK encoding genes, the most frequent of which were FLT4, ROS1, EPH7, ERBB2, EGFR, RET, FGFR3 and FGFR4, ii) the majority of the observed variants were variants of unknown significance (VUS), being only 10% present in the COSMIC database, iii) somatic mutations in the RET proto-oncogene were identified in 4 colon cancer patients (10%), iv) only one RET variants were apparently active and able to stimulate anchorage-dependent proliferation of HEK293 cells, resembling MEN2A-like mutations, since they efficiently generate RET dimers in transfected HEK293 cells. These issues will be commented more in detail below.

Upon re-analysis of the data reported in Oliveira et al. (under review) we have focused on genes encoding RTKs, and have observed that RTKs are potentially involved in the initiation and/or progression of at least 75% of colon cancer. In fact, 28 patients out of 37 presented variants in at least one gene encoding RTKs. Co-occurrence of variants in different RTK genes was observed in 20 tumors out of 37 (54%), with several samples showing variants in multiple RTK genes simultaneously (CC27, 11 genes; CC34, 13 genes; CC12, 8 genes), which raised the issue of whether these variants were indeed functionally relevant.

An important issues raised by this work is the finding that the vast majority of variants identified in RTK genes are VUS. Only few variants could be easily categorized as known pathogenic mutations, such as those present in the COSMIC database [FLT4 (G1131S, R1041Q, E1026K), EGFR (R669*, W731*), ERBB2 (S310F), ERBB4 (R847H), IGF1R (I1151M), EPHB1 (A922T) genes]. This observation raises the issue of the clinical significance of the variants identified, and consequently of the use that oncologists should do to patients with colon cancer that carry VUS in RTK genes. Although 30/101 of the variants identified in this study promoted amino acid changes within the catalytic domains of the corresponding RTKs, the ability to provide a clinical interpretation of VUS represents a daunting task because clinical or experimental data that aid interpretation are rarely available. The only valid alternative for clinicians is to evaluate significance by the use of computational tools that predict the biologic effects of novel variants [33] on the basis of available literature. In this manuscript we have used two different prediction algorithms, SIFT and Polyphen2, to define a variant as pathogenic. However the oncogenic role of VUS remains unclear.

The choice of RET for subsequent validation of RTK variants was due to the fact that, at difference with what happens in thyroid and lung cancer, the involvement of RET proto-oncogene to the development of colon cancer is still debated. On one hand, it has been proposed that RET is a potential tumor suppressor gene in colorectal cancer [34]. RET is expressed in normal mucosa, and its expression is lost in adenomas and adenocarcinomas because of an aberrantly methylation of CpG islands within its promoter [34]. Notably, RET promoter methylation is associated with poor prognosis in stage II and III colorectal cancer patients [35]. Moreover, although RET mutations (V145G, R360W or G593E) in colorectal cancer have been identified [36] and are reported in the COSMIC database, these mutations apparently inactivate RET [34]. On the other hand, Le Rolle and co-workers have recently reported on the identification and characterization of RET fusions that juxtapose the C-terminal RET kinase domain to the N-terminal domain of CCD6 or NCOA4 (CCDC6-RET and NCOA4-RET fusions) in advanced colorectal cancer. Importantly, a patient carrying the CCDC6-RET fusion showed a therapeutic response when treated with the RET kinase inhibitor regorafenib [37].

In this manuscript, we have investigated whether the RET variants identified in colon cancer patients are able to confer pro-tumorigenic properties to epithelial cells. We observed that HEK293 cells expressing RET-G533C, but not the same cells expressing RET-WT or RET-P1047S, duplicated with an increased rate in monolayer and in clonogenic assays and promoted migration compared with control cells. Accordingly, the active RET mutant detected in colon cancer patients (G533C) may resemble those described in MEN2A patients, of which the C634R is the prototype [38]. In fact, RET mutant G533C was particularly efficient in generating RET dimers in transfected HEK293 cells, suggesting that the oncogenic mechanism whereby this RET mutant contributes to colon cancer development occurs through an increase in the amount of covalently linked dimers induced by the presence of the unpaired cysteine caused by the mutations. Such increased amount of dimers would turn into increased activity of RET receptors (detected as increased RET phosphorylation), which in turn would cause activation of the MAPK signalling pathway (detected as increased ERK1/2 phosphorylation).

It is of note that the RET inhibitor vandetanib abolished phosphorylation of RET Y1062 and of ERK1/2 Y202/T204 in parallel with a marked reduction of the proliferative effects induced by the RET G533C variant.

These results indicate that RET mutant G533C, but not wild type or P1047S RET expressed at similar level, is able to stimulate proliferation and migration of human epithelial cells in culture. In addition, the finding that the sample with RET G533C variant presented variants in the downstream effectors like KRAS or MAPK whereas the samples that carries the RET P270L or the RET P1047S variants presented also variants in other genes (KRAS, AKT1 and BRAF, PTEN, AKT1 and FGFR3, respectively) indicated that the RET G533C represents a real gain-of-function mutation.

## Conclusions

In conclusion, we have re-analysed NGS data generated on 37 colon cancer patients by use of Comprehensive Cancer Panel, focusing on genes encoding RTKs. Overall, we have observed 101 different potentially damaging variants distributed across 31 RTK genes in 28 patients, suggesting that the remaining tumors represent a subset of colon cancer that may develop through pathways that are independent of RTK signalling. However, only few variants could be unambiguously categorized as pathogenic mutations, being the vast majority of RTK variants identified in this study VUS, whose clinical significance remains unclear. This is best exemplified by the validation analysis performed on 2 different RET variants. The results presented here indicate that RET variant G533C is clearly oncogenic whereas RET variant P1047S is not. Yet, the identification of gain-of-function RET variants in colon cancer patients provides novel data for the involvement of this RTK in the development of colon cancer and identifies RET as a novel putative target for targeted therapy.

## Additional files

Additional file 1: **Table S1.** Clinical-pathological characteristics of the 37 colon cancer patients included in the study. (XLSX 12 kb)

Additional file 2: **Table S2.** Summary of the variants identified in RTKs. (XLSX 16 kb)

Additional file 3: **Table S3.** List of mutated RTKs in each patient included in the study. (XLSX 11 kb)

Additional file 4: **Table S4.** Sequencing metrics of the identified RET mutations. (XLSX 10 kb)

Additional file 5: **Table S5.** List of mutations identified in CC20 after the next generation sequencing analysis pipeline. (XLSX 17 kb)

## Abbreviations

CCP: Comprehensive Cancer Panel
CRC: Colorectal cancer
HEK293: Human embryonic kidney cells 293
HSNCC: Head and neck squamous-cell carcinoma
IGV: Integrated Genomic Viewer
MAPK,: Mitogen-activated protein kinase
NGS: Next Generation Sequencing
PTKs: Protein tyrosine kinases
RTKs: Receptor-type tyrosine kinases
SDS-PAGE: Sodium Dodecyl Sulphate - PolyAcrylamide Gel Electrophoresis;
SIFT: Scale-invariant feature transform
UICC: Union for International Cancer Control
VUS: Variants of unknown significance
