# Correction to: Next-generation sequencing analysis of receptor-type tyrosine kinase genes in surgically resected colon cancer: identification of gain-of-function mutations in the RET proto-oncogene

**DOI:** 10.1186/s13046-018-0776-5

**Published:** 2018-06-01

**Authors:** Duarte Mendes Oliveira, Katia Grillone, Chiara Mignogna, Valentina De Falco, Carmelo Laudanna, Flavia Biamonte, Rosa Locane, Francesco Corcione, Massimiliano Fabozzi, Rosario Sacco, Giuseppe Viglietto, Donatella Malanga, Antonia Rizzuto

**Affiliations:** 1 Department of Experimental and Clinical Medicine, University Magna Graecia of Catanzaro, Campus Salvatore Venuta -Viale Europa, 88100 Catanzaro, Italy; 2 Department of Health Sciences, University Magna Graecia of Catanzaro, Campus Salvatore Venuta - Viale Europa, 88100 Catanzaro, Italy; 3 Department of Medical and Surgical Sciences, University Magna Graecia of Catanzaro, Campus Salvatore Venuta - Viale Europa, 88100 Catanzaro, Italy; 4 Department of Molecular Medicine and Medical Biotechnologies, University Federico II, Naples, Italy; 5 UOC Chirurgia Generale, Azienda Ospedaliera dei Colli, Naples, Italy

## Correction

In the publication of this article [1], there is an error in Fig. 7. The minus and plus signals are inverted which impairs understanding of the results described.

Figure 7 should instead be read as indicated in this correction.

**Fig. 7:**
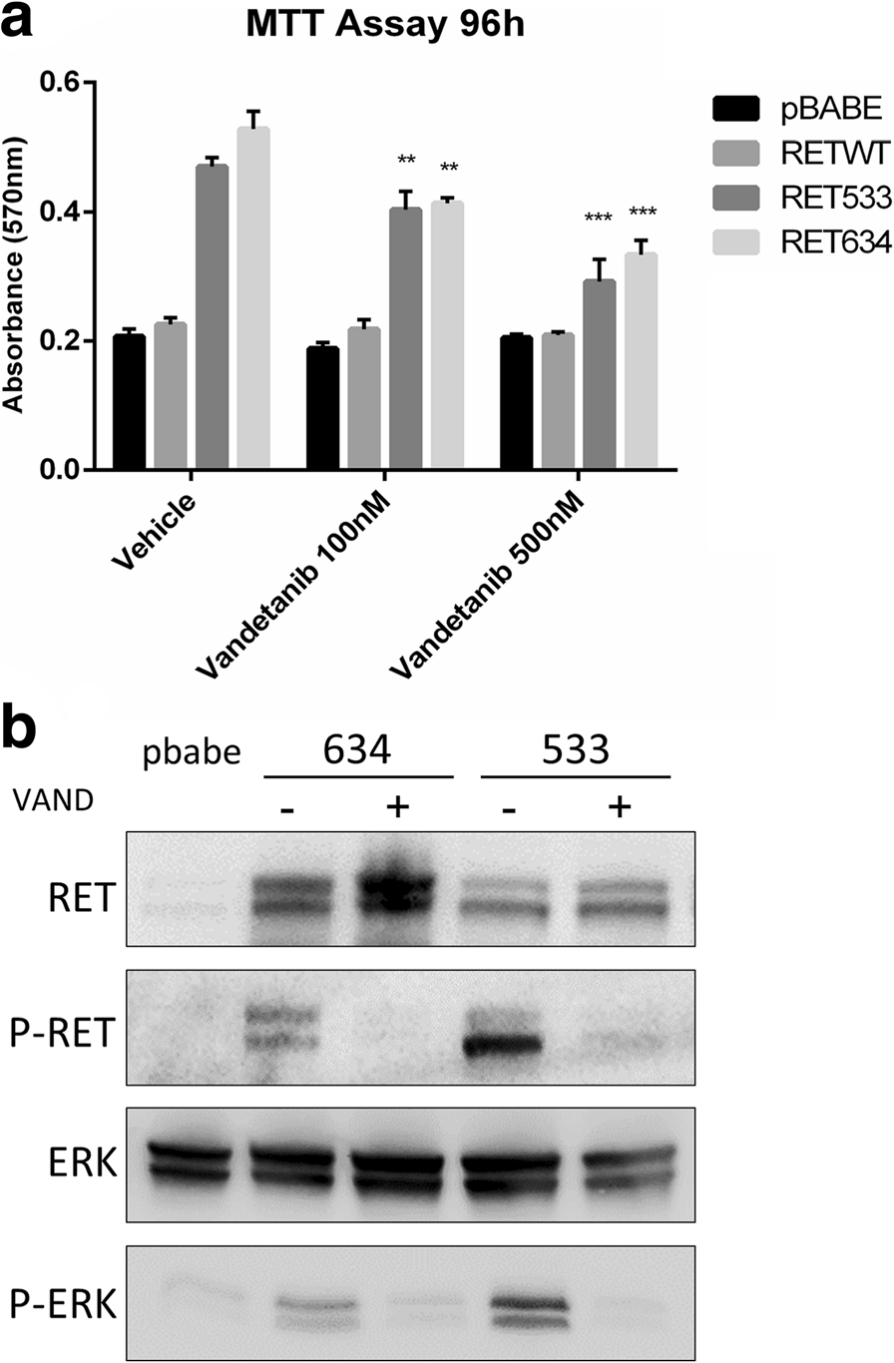
From: Next-generation sequencing analysis of receptor-type tyrosine kinase genes in surgically resected colon cancer: identification of gain-of-function mutations in the RET proto-oncogene. The RET-G533C mutant is inhibited by vandetanib. **a**. MTT assay was performed in the presence of two different concentrations of vandetanib. HEK293 cells expressing pBabe were used as negative control and HEK293 cells expressing RET-C634R mutant were used as positive control. Values are shown as bar graphs and all results are the average of experiments performed in triplicate. Statistical significance compared with vehicle was evaluated by Two-Way ANOVA (with multiple comparison Dunnet’s test) (*n* = 3; **p < 0.01; ***p < 0.001). **b**. Immunoblot analysis of RET/MAPK pathway (with anti-phosphoY1062 RET, anti-RET, anti phospho-Y202T204 ERK1/2, anti-ERK1/2 antibodies) of HEK293 cells transfected with RET-G533C mutant treated with vehicle or vandetanib (500 nM) for 2 h. HEK293 cells transfected with RET-634 mutant were used as control

This has now been included in this erratum.
